# The Gut-Joint Connection: Microbiome’s Role in Rheumatic Disease

**DOI:** 10.5152/ArchRheumatol.2025.25192

**Published:** 2025-12-01

**Authors:** Nilay Şahin, Ender Salbaş

**Affiliations:** Department of Physical Medicine and Rehabilitation, Balıkesir University Faculty of Medicine, Balıkesir, Türkiye

**Keywords:** Arthritis, autoimmune disease, dysbiosis, gut-joint axis, intestinal permeability, microbiome, rheumatology, Th17, Treg balance

## Abstract

The human gut microbiome is a pivotal regulator of systemic immunity and a central factor in the pathogenesis of rheumatic diseases. An imbalance in this microbial community, known as “dysbiosis,” can trigger and perpetuate autoimmune responses through the “gut-joint axis.” A key mechanism underpinning this connection is increased intestinal permeability (“leaky gut”), which facilitates the translocation of microbial products like lipopolysaccharide into the systemic circulation, thereby provoking chronic inflammation. Concurrently, dysbiosis disrupts the critical homeostatic balance between pro-inflammatory Th17 cells and regulatory T cells, an immunological hallmark of conditions such as rheumatoid arthritis (RA), ankylosing spondylitis, and systemic lupus erythematosus (SLE).

Specific microbial signatures, including the expansion of *Prevotella copri* in RA and *Ruminococcus gnavus* in SLE, are emerging as potential diagnostic biomarkers. This deeper understanding is paving the way for innovative therapeutic strategies. Interventions aimed at modulating the gut microbiota, such as targeted diets, probiotics, prebiotics and fecal microbiota transplantation, represent a promising frontier for the personalized management of rheumatic diseases. This review explores the foundational mechanisms linking the microbiome to autoimmunity and discusses the clinical potential of harnessing the gut-joint axis to improve patient outcomes.

**Main Points**

- In the pathogenesis of rheumatic diseases, an imbalance in the gut microbiota (dysbiosis) is not just a consequence but also a central triggering factor.
- Increased intestinal permeability (“leaky gut”) is a key mechanism leading to the entry of microbial products (e.g., lipopolysaccharide) into the systemic circulation and triggering chronic inflammation.
- The microbiome modulates the sensitive balance between pro-inflammatory (Th17) and anti-inflammatory regulatory T cells in the immune system, and disruption of this balance intensifies autoimmune responses.
- The “gut-joint axis” concept explains how events in the gut directly affect joint health through systemic inflammation, immune cell migration, and microbial metabolites.
- Targeting the microbiome offers significant potential for innovative and personalized treatment strategies, including diet, probiotics, prebiotics and fecal microbiota transplantation (FMT).

## Introduction and Basic Mechanisms

The human body harbors an ecosystem comprising human cells alongside bacteria, archaea, viruses, and fungi, termed the human microbiome.^1^^-^^3^ The majority of the microbial load resides in the large intestine, hosting >1500 species across >50 phyla, with *Bacteroidetes *and *Firmicutes* constituting the bulk of adult gut bacteria.^1^^,^^4^^,^^5^ The microbiota maintains a symbiotic “eubiosis” balance with the host, performing metabolic, protective, and immune-regulatory functions: fermentation of fibers, vitamin synthesis, and short-chain fatty acid (SCFA) production; prevention of pathogen colonization and support of barrier integrity; and education of mucosal immune responses are among the primary ones.^1^^,^^3^^,^^6^^-^^8^ These mutual interactions have heightened interest in the microbiome’s role in the etiology of autoimmune and rheumatic diseases.^1^^,^^2^^,^^8^^-^^13^

### Barrier Integrity and “Leaky Gut”

**Figure 1.**
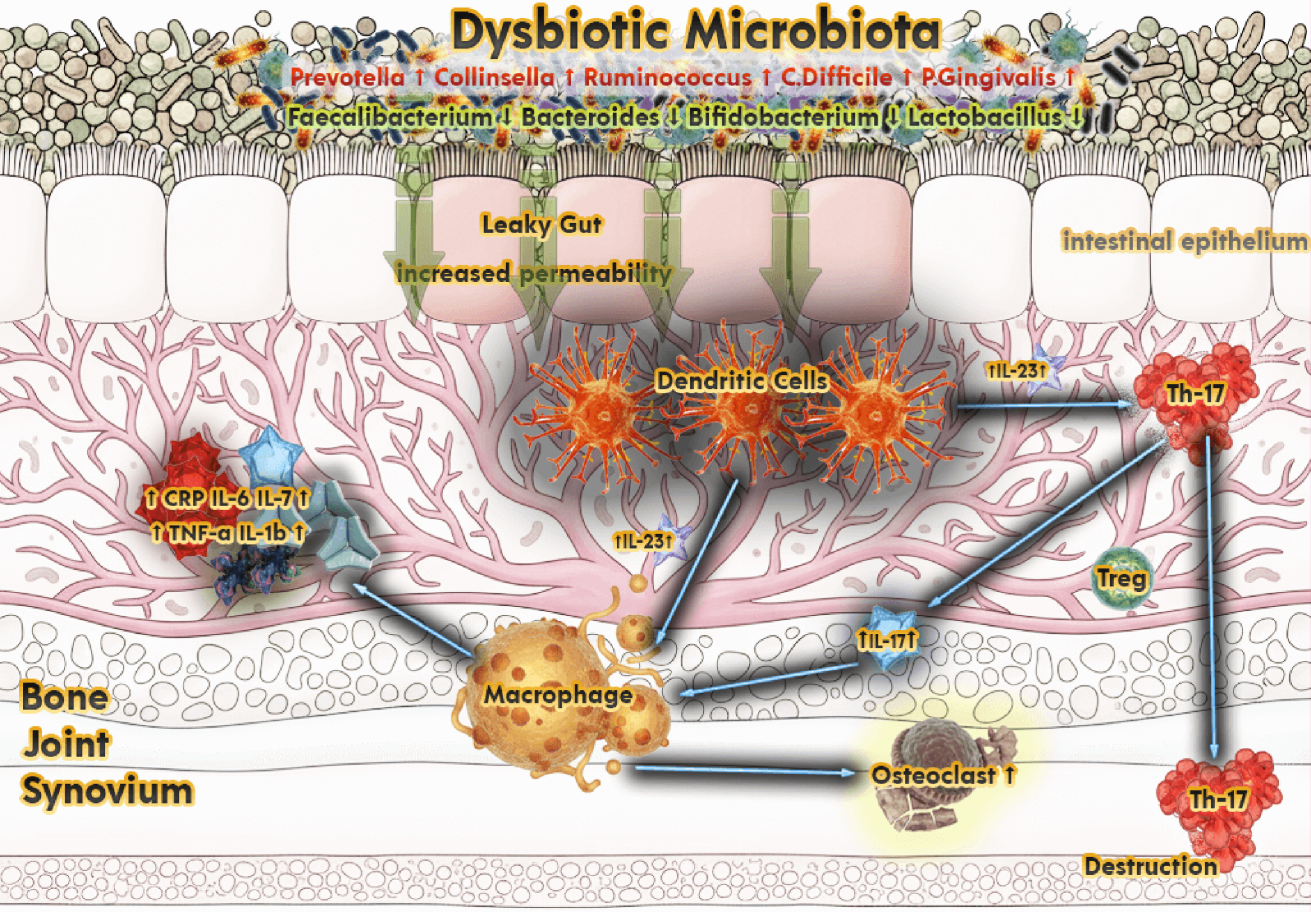
Dysbiotic gut microbiota and the gut-joint axis mechanism in rheumatic diseases. Schematic representation of the gut-joint axis linking intestinal dysbiosis to systemic inflammation and joint destruction in rheumatic diseases. The illustration depicts the intestinal lumen densely populated by microbial communities. Dysbiosis—characterized by increased *Prevotella, Collinsella, Ruminococcus, Clostridioides* difficile, and *Porphyromonas gingivalis* alongside decreased *Faecalibacterium*, *Bifidobacterium*, and L*actobacillus*—leads to disruption of the intestinal epithelial barrier (“leaky gut”). This increased permeability allows microbial products such as lipopolysaccharides (LPS) to enter the lamina propria and circulation, activating dendritic cells and macrophages via pro-inflammatory cytokines (e.g., IL-6, IL-1β, TNF-α, IL-23). The resulting skew toward Th17 differentiation (via IL-23 and IL-17 signaling) and reduced regulatory Tactivity promotes osteoclastogenesis, synovial inflammation, and bone destruction in joints. The figure integrates intestinal, immune, and skeletal compartments, illustrating the central mechanistic pathway by which gut dysbiosis contributes to rheumatic disease pathogenesis.

Tight junctions in the intestinal epithelium limit passage from the lumen to systemic circulation; disruption of zonulin-mediated regulation increases permeability, allowing microbial products such as lipopolysaccharide (LPS) to leak and elevate tumor necrosis factor-α (TNF-α)/Interleukin-6 (IL-6) production via the TLR4–MyD88 pathway, thereby amplifying systemic inflammation.^3^^,^^9^^,^^14^^-^^20^ In ankylosing spondylitis (AS), epithelial and vascular barrier disruption with elevated zonulin levels has been reported; similarly, increased permeability consistent with dysbiosis has been noted in rheumatoid arthritis (RA) and systemic lupus erythematosus (SLE).^21^^-^^25^ This barrier dysfunction contributes to microbial product leakage and peripheral immune tolerance breakdown, supporting autoantigenic stimulation and heightened immune signaling^9^ (Figure 1).

### Helper T 17/Regulatory T Balance

The gut microbiota regulates the balance between helper T 17 (Th17) and regulatory T (Treg) cells, which play a central role in the pathogenesis of rheumatic diseases.^14^^,^^26^^-^^29^ The Th17 cells produce IL-17 to promote autoimmune inflammation, whereas Treg cells maintain immune tolerance. Gut dysbiosis disrupts this balance. For instance, segmented filamentous bacteria colonization activates Th17 cells, whereas certain commensals like *Bacteroides fragilis* can restore equilibrium.^14^^,^^28^^,^^30^ The SCFAs, particularly butyrate, support Treg cell formation, exerting immunomodulatory effects and limiting autoimmune responses. This Th17/Treg imbalance is a fundamental feature of many autoimmune diseases, including RA, SLE, and AS.^14^^,^^28^^,^^31^^-^^36^

### Microbial Metabolites

Molecules produced by the microbiota, such as SCFAs, tryptophan metabolites, and bile acids, profoundly influence host physiology and immune responses.^6^^,^^12^^,^^37^ Short-chain fatty acids, generated from dietary fiber fermentation, include butyrate, which possesses potent anti-inflammatory properties. Butyrate mitigates inflammation and bone loss in conditions like osteoarthritis (OA), AS, and RA by regulating Treg cells and improving gut barrier function.^7^^,^^12^^,^^31^^,^^34^^,^^36^^-^^40^ Indoles from tryptophan metabolism act via the aryl hydrocarbon receptor (AhR) in immune modulation.^18^^,^^41^^-^^43^ In patients with RA, anti-inflammatory tryptophan metabolites decrease, whereas pro-inflammatory metabolites increase.^18^

### Molecular Mimicry

This mechanism triggers autoimmunity through the structural resemblance between microbial antigens and host proteins.^38^^,^^44^^,^^45^ For example, enolase produced by *Porphyromonas* *gingivalis*, which is associated with RA, can elicit autoimmunity against human enolase.^44^^,^^46^ Similarly, strains of *Ruminococcus* *gnavus* linked to SLE may initiate cross-reactive immune responses against host double-stranded DNA (dsDNA).^14^ *Aggregatibacter actinomycetemcomitans* induces citrullination, linking periodontal infection to RA autoimmunity.^45^ This illustrates how even normal microbial community members can prompt the immune system to attack the host tissues.

### Gut-Joint Axis

This concept denotes a bidirectional relationship between the gut microbiota and joint health.^5^^,^^46^^,^^47^ Gut microbiota dysbiosis contributes to the development of various joint diseases, including RA, AS, psoriatic arthritis (PsA), and OA.^5^^,^^48^^-^^51^ The axis functions through mechanisms like systemic inflammation from leaky gut, circulation of microbial metabolites like SCFAs to joints, and migration of gut-activated immune cells to joints.^7^^,^^19^^,^^52^^,^^53^ In germ-free (microbe-free) animal models, particularly *HLA-B27* transgenic mice, the prevention of gut and joint inflammation strongly supports the central role of the microbiota in these diseases.^17^^,^^44^^,^^54^^,^^55^

## Inflammatory Arthritides and Microbiome Dysbiosis

Evidence is mounting that imbalances in the gut and oral microbiota play critical roles in the pathogenesis of inflammatory arthritides. Microbiome dysbiosis is strongly associated with the onset and progression of these diseases (Table 1).^26^^,^^45^^,^^50^

### Rheumatoid Arthritis and Microbiome Dysbiosis

In RA, autoimmune responses are thought to originate from the mucosal surfaces.^28^ Patients with RA exhibit a profile in both oral and gut microbiota characterized by reduced beneficial bacteria and increased pro-inflammatory bacteria.^9^

#### Gut Dysbiosis: Increased *Prevotella copri* and Decreased *Faecalibacterium prausnitzii*:

In RA patients, particularly those with new-onset RA, an increase in *Prevotella copri* has been observed.^28^^,^^30^ *P. copri* is identified as a pro-inflammatory bacterium that exacerbates arthritis in experimental models and promotes T-cell differentiation into Th17 cells, fueling systemic inflammation.^27^^,^^28^^,^^36^^,^^56^^,^^57^ Conversely, beneficial bacteria like *Faecalibacterium prausnitzii*, which possess anti-inflammatory properties and produce butyrate, are decreased.^15^^,^^25^^,^^58^ **Faecalibacterium* prausnitzii* has been shown to alleviate inflammatory arthritis in experimental models.^27^^,^^50^^,^^59^ This imbalance can lead to impaired gut barrier function and systemic inflammation.^10^^,^^15^^,^^20^^,^^26^

#### Oral Microbiome: Role of *Porphyromonas gingivalis*, Citrullination, and anti-citrullinated protein Antibodies:

The oral microbiome, particularly through periodontal diseases, plays a significant role in RA pathogenesis.^60^^,^^61^ *Porphyromonas gingivalis*, a key pathogen in periodontal disease, is associated with RA onset.^44^ This bacterium citrullinates proteins via its unique microbial peptidylarginine deiminase enzyme, triggering the production of anti-citrullinated protein antibodies (ACPA).^44^^,^^54^^,^^61^ These ACPAs cross-react with human citrullinated peptides, thereby initiating autoimmunity.^44^ The ACPA-positive individuals have an increased risk of RA, often accompanied by a dysbiotic oral microbiome with elevated *levels of P. gingivalis*.^9^^,^^38^^,^^45^

### Axial Spondyloarthritis, Psoriatic Arthritis, and Microbiome Dysbiosis

Axial spondyloarthritis (AxSpA) and PsA are inflammatory arthritides closely linked to the gut microbiome. There is a strong clinical and genetic connection between these diseases and inflammatory bowel disease (IBD).^22^^,^^62^^,63^ Dysbiotic features observed in patients with IBD, such as reduced phylogenetic diversity, lower Firmicutes proportion and particularly decreased *F. prausnitzii*, are also seen in patients with PsA.^9^^,^^34^^,^^54^^,^^62^^,^^63^

Subclinical gut inflammation is common in patients with AxSpA and PsA. *HLA-B27* transgenic rats do not develop inflammation under germ-free conditions, suggesting that commensal bacteria are triggers.^17^^,^^36^^,^^44^^,^^55^ The IL-23/IL-17 signaling pathway plays a central role in AxSpA pathogenesis, and microbial signals can activate this pathway.^35^^,^^36^^,^^39^^,^^62^ Microbiota dysbiosis drives immune dysregulation by shifting T cell differentiation toward a Th17-dominant phenotype and increasing IL-17 production.^27^^,^^64^ Specific microbial differences have been identified in both AxSpA and PsA;

- **Axial spondyloarthritis:** Reduced bacterial diversity^39^^,^^44^^,^^57^ with increased abundance of genera such as *Klebsiella*, *R. gnavus,* and *Streptococcus* and decreased *abundance of Dialister* and *Faecalibacterium*.^39^^,^^44^^,^^55^^,^^57^^,^^65^ Additionally, *Proteobacteria* and *Pasteurellaceae* levels were normalized after TNF inhibitor therapy.^66^
- **Psoriatic arthritis: **Reduced bacterial diversity, similar to IBD.^9^^,^^34^^,^^54^^,62^ Increased *Actinobacteria* phylum and decreased *Rikenellaceae* family.^49^^,^^63^

## Evidence in Other Diseases

### Systemic Lupus Erythematosus

Patients with SLE exhibit marked changes in the gut microbiota, with a decreased abundance of *Firmicutes* and an increased abundance of *Bacteroidetes*.^28^ Notably, *R.* *gnavus* abundance increases during active disease periods and correlates with disease activity.^9^^,^^14^^,^^28^^,^^44^ Antibodies against *R. gnavus* may cross-react with host DNA, triggering anti-dsDNA antibody responses.^14^^,^^28^ Increased intestinal permeability and abnormalities in Type I interferon production are key contributors to SLE pathogenesis.^9^^,^^14^^,^^34^^,^^67^

### Sjögren’s Syndrome

Sjögren’s syndrome (SS) is associated with distinct changes in both oral and intestinal microbiota.^13^^,^^68^^,^^69^ Oral dysbiosis in the oral microbiota^45^^,^^70^ and severe dysbiosis in the gut microbiota are common and correlate with systemic disease activity.^25^^,^^68^^,^^69^ Similar to RA and SLE, SS shows common alterations like decreased anti-inflammatory butyrate-producing bacteria (e.g., *Faecalibacterium*) and increased pro-inflammatory bacteria (e.g., *Streptococcus*).^25^

### Systemic Sclerosis

In systemic sclerosis (SSc), a unique gut microbial composition is identified, with decreased protective butyrate-producing bacteria and increased harmful genera like *Fusobacterium*.^25^^,^^37^ Increased intestinal permeability may also play a role in the pathogenesis of SSc.^25^^,^^37^^,^^71^

### Vasculitides and Metabolic Diseases

Children with IgA vasculitis (HSP) have been found to have increased abundance of *Fusobacteria* and decreased abundance of *Firmicutes*.^72^ Behçet’s disease shows changes toward increased abundance of Bifidobacteria in the gut.^21^ Osteoarthritis, traditionally considered non-inflammatory, is now linked to low-grade systemic inflammation (metaflammation) and gut microbiota. Leaked LPS from the gut can initiate obesity-related inflammation, worsening OA progression.^19^^,^^46^^,^^73^ In Gout, the microbiome’s impact on purine metabolism and uric acid levels is under investigation, with reduced microbial diversity observed in patients.^25^^,^^74^

## Factors Influencing the Microbiome: Diet, Lifestyle, and Medications

Understanding the factors that shape the composition and function of the microbiome can offer new strategies for disease management. These factors primarily include diet, lifestyle, and medications.^2^^,^^12^^,^^75^

### Diet and Metabolism: Primary Shapers of the Microbiome

Diet is one of the most potent environmental factors that can rapidly alter the gut microbiota composition.^2^^,^^30^^,^^76^ Western-style diets that are low in fiber and high in fat can lead to pro-inflammatory changes by reducing butyrate-producing bacteria.^28^^,^^39^^,^^42^ In contrast, fiber-rich diets (prebiotics) and fermented foods (probiotics) enhance SCFA production and modulate the immune system, providing benefits.^11^^,^^12^^,^^31^^,^^36^^,^^68^ Particularly, butyrate can inhibit systemic inflammation by activating Treg cells and strengthening the gut barrier.^32^^,^^45^^,^^72^^,^^77^ Strategies such as fecal microbiota transplantation (FMT) also hold promise for correcting dysbiosis.^13^^,^^14^^,^^44^^,^^75^^,^^76^^,^^78^

### Lifestyle and Medications: Interactions on the Microbiome

Smoking can contribute to oral microbiome dysbiosis,^33^^,^^44^ while chronic stress adversely affects the gut-brain axis.^43^^,^^47^^,^^79^^,^^80^ Medications have a strong effect on the microbiome. Antibiotics are the most potent agents that alter microbiota composition and diversity.^2^^,^^8^^,^^18^^,^^28^^,^^30^^,^^54^ Nonsteroidal anti-inflammatory drugs and proton pump inhibitors can damage the gut barrier and alter the microbiome composition, contributing to inflammation.^22^^,^^39^^,^^54^^,^^81^^-^^83^ Disease-modifying antirheumatic drugs and biologics can modulate the microbiome. For example, methotrexate and sulfasalazine may reduce the levels of *B. fragilis* and *Enterobacteriaceae* in the gut.^26^ The TNF inhibitors can increase microbiota diversity in patients with AS, approximating a healthy profile.^17^^,^^36^^,^^39^^,^^66^^,^^71^ Janus kinase inhibitors, such as tofacitinib, are associated with significant changes in certain gut bacterial lineages in patients with PsA and may exert therapeutic effects by modulating plasma metabolites and the gut microbiota.^23^^,^^63^

## Microbiome Research in Special Populations, Pain, Virome, and Cardiovascular Comorbidities

Microbiome research extends beyond bacteria to include viruses (virome) and fungi (mycobiome), which broadens the understanding of disease pathogenesis.

The microbiome shaped during early life is critical for immune system development.^54^ Marked dysbiosis in both oral and gut microbiota has been observed in childhood autoimmune diseases, such as juvenile idiopathic arthritis (JIA). Notably, reductions in beneficial butyrate-producing bacteria, such as *F. prausnitzii*, highlight the potential of microbiome-based approaches in JIA treatment.^84^

In chronic pain conditions like fibromyalgia (FMS), the gut microbiome plays a critical role in pathogenesis via the “gut-brain axis.”^47^^,80^ Metabolites produced by gut microbes, such as SCFAs, can directly or indirectly influence the central nervous system and modulate pain perception. This may be a common denominator in comorbid conditions like FMS, chronic fatigue syndrome, and irritable bowel syndrome.^47^^,^^80^

The unseen aspects of the microbiome, including the virome and mycobiome, also contribute to autoimmunity. Viruses like Epstein-Barr virus may initiate autoimmune responses through molecular mimicry.^14^^,^^44^^,^^45^ Similarly, changes in gut fungal composition (mycobiome) are thought to play a role in diseases like IBD and modulate immune responses.^15^^,^^76^

### Cardiovascular Comorbidities in Rheumatic Diseases

Rheumatic diseases often co-occur with cardiovascular diseases due to systemic effects of chronic inflammation.^80^ This systemic inflammation is closely linked to endothelial cell activation and atherosclerosis.^37^ The ability of the microbiome to modulate inflammatory responses is a key mechanism linking rheumatic diseases and cardiovascular comorbidities.

### Role of Animal Models in Understanding Mechanisms and Limitations in Translation to Human Studies

Animal models, particularly gnotobiotic models (germ-free or colonized with known microorganisms), are indispensable for studying host-microorganism interactions.^54^ For instance, destabilization of the medial meniscus mouse models demonstrated that gut microbiome dysbiosis accelerates OA progression.^19^ Experiments using “humanized” germ-free SKG mice with human gut microbiota revealed that protein tyrosine phosphatase non-receptor type 2 haploinsufficiency variably exacerbates mannan-induced arthritis when colonized with feces from RA patients, showing increased T cell-driven inflammation in the joints.^24^ Similarly, dextran sulfate sodium-induced colitis models and collagen-induced arthritis models are used to examine the effects of microbiota on inflammation.^50^^,^^85^

However, translating the findings from animal models to humans faces significant limitations. The most prominent constraint is the difference in bacterial composition between humans and animals.^19^ Additionally, host genetics and environmental factors may not fully reflect the complex disease phenotypes seen in humans.^75^ Thus, while animal models are critical for mechanistic insights, rigorous human studies are essential for clinical application.^75^

## Clinical Horizons and Conclusion

The microbiome has emerged as a potential biomarker source for disease diagnosis, prognosis prediction, and treatment response determination. For example, the microbiome composition in patients with FMS can be distinguished from that in healthy individuals with high accuracy.^56^ Distinct microbiota and metabolite profiles have been identified among different uveitis types.^21^ Specific bacterial species like *Bacteroides caecimuris, Mediterranea massilliensis, Bacteroides coprophilus, Clostridium_sp_7_3_54FAA* and *Bifidobacterium* *bifidum *may aid in differentiating patients with SS from healthy individuals and correlate with disease severity.^13^ In patients with AS, species such as *R*. *gnavus *could serve as markers, with TNF inhibitor therapy shifting the microbiome toward a healthy profile.^39^^,^^66^

However, most current studies are correlational and insufficient to prove causality.^13^^,^^56^ Methodological variations and population heterogeneity also limit generalizability.^13^^,^^39^^,^^56^

Therapeutic strategies, including dietary interventions, prebiotics, probiotics, postbiotics (SCFAs), and FMT, show promise. Probiotics have demonstrated the potential to improve inflammation and symptoms.^14^^,^^15^^,^^47^^,^^50^^,^^67^^,^^79^^,^^86^ SCFAs, such as butyrate, have been shown to reverse bone loss and reduce inflammation in animal models.^2^^,^^36^^,^^47^^,^^66^ The FMT, proven effective in Clostridioides difficile infections, also yields encouraging results in autoimmune diseases such as SLE and RA.^13^^,^^14^^,^^28^^,^^87^

The greatest challenges in fully harnessing the microbiome in clinical practice include distinguishing causality from correlation, overcoming inter-study heterogeneity, and understanding each individual’s unique microbiome structure.^13^^,^^43^^,^^49^^,^^56^ In the future, personalized treatment strategies that integrate microbiota profiling, host genetics, and other factors could redefine therapeutic paradigms for rheumatic and other chronic diseases.^2^^,^^47^

In conclusion, the gut microbiota is an indispensable player in human health and disease. As knowledge in this field translates to clinical practice, it holds immense potential for the diagnosis, prognosis, and treatment of diseases. Future research should facilitate the integration of microbiome-based therapies into clinical routines using personalized and targeted strategies. The microbiome opens doors to a new era of a healthier future in medicine.

## Figures and Tables

**Table 1.** Summary of Microbiota Changes in Rheumatic Diseases

**Disease**	**Increased Bacteria in Disease**	**Decreased Bacteria in Disease**	**Potential Mechanisms and Effects**
Rheumatoid arthritis (RA)	*Prevotella copri, Porphyromonas gingivalis *(oral)*, Aggregatibacter actinomycetemcomitans*	*Faecalibacterium prausnitzii, Roseburia, Bifidobacterium *	Molecular mimicry (citrullination, ACPA production), Th17 increase, increased intestinal permeability, systemic inflammation
Ankylosing spondylitis (AS)	*Ruminococcus gnavus, Klebsiella pneumoniae, Staphylococcus, Escherichia, Streptococcus*	*Dialister, Lachnoclostridium, Oscillibacter, Faecalibacterium, Roseburia *	IL-23/IL-17 activation, leaky gut, subclinical gut inflammation, HLA-B27 association
Psoriatic arthritis (PsA)	*Actinobacteria (Megamonas), Howardella, Methanobrevibacter, Sutterella *	*Rikenellaceae, Eubacterium brachy, Bacteroidales *	IBD-like dysbiosis, IL-17 increase, reduced bacterial diversity, modulation with tofacitinib
Systemic lupus erythematosus (SLE)	*Ruminococcus gnavus, Bacteroides thetaiotaomicron, Ruminococcus intestinalis, Akkermansia muciniphila *	*Firmicutes *(general decrease),* Odoribacter splanchnicus, Bacteroides fragilis*	Molecular mimicry (dsDNA cross-reaction), Th17/Treg imbalance, leaky gut, IFN-I dysregulation
Sjögren’s syndrome (SS)	*Lactobacillus salivarius, Bacteroides fragilis, Ruminococcus gnavus, Streptococcus parasanguinis *	*Faecalibacterium, Haemophilus parainfluenzae, Roseburia *	Oral/intestinal dysbiosis, Th17 increase, anti-inflammatory SCFA decrease, systemic activity association
Systemic sclerosis (SSc)	*Fusobacterium, Ruminococcus, Erwinia, Eggerthella lenta, Clostridium bolteae *	*Bacteroidetes *(general decrease), *Faecalibacterium, Clostridium*	Th17 induction, homocysteine production, leaky gut, vasculopathy and inflammation
Osteoarthritis (OA)	*Firmicutes *(increase),* Bilophila, Desulfovibrio, Streptococcus*	*Roseburia, Bacteroidetes *(general decrease),* Bacteroides acidifaciens *(in non-obese)	LPS leakage, metaflammation, SCFA imbalance (acetic acid increase), joint destruction
Gout disease	*Proteobacteria* (increase),* Verrucomicrobia*	*Firmicutes *(decrease),* Bifidobacterium*	Uric acid metabolism disruption, purine metabolism abnormalities, alpha diversity decrease

## Data Availability

The data that support the findings of this study are available on request from the corresponding author.
